# Supplementary material for: Potentiating TMEM16A does not stimulate airway mucus secretion or bronchial and pulmonary arterial smooth muscle contraction
Source: FASEB Bioadv. 2020 Jul 1;2(8):464–77. doi: 10.1096/fba.2020-00035 (PMC7429354; doi:10.1096/fba.2020-00035)
Supplement: Supplementary file 1 — Supplementary Material [file FBA2-2-464-s001.pdf]

SUPPLEMENTAL INFORMATION

Potentiating TMEM16A does not stimulate airway mucus secretion or bronchial and pulmonary arterial smooth muscle contraction

Henry Danahay[1], Roy Fox[2], Sarah Lilley[2], Holly Charlton[2], Kathryn Adley[2], Lee Christie[3], Ejaz Ansari[3], Camille Ehre[4], Alexis Flen[4], Michael J. Tuvim[5], Burton F. Dickey[5], Colin Williams[6], Sarah Beaudoin[6], Stephen P Collingwood[1], Martin Gosling[1,2]

[1] Enterprise Therapeutics Ltd. Science Park Square, Brighton, BN1 9SB, UK

[2] School of Life Sciences, University of Sussex, Brighton, BN1 9QG, UK

[3] REPROCELL Europe Ltd. West of Scotland Science Park, Acre Road, Glasgow, G20 0XA UK

[4] Marsico Lung Institute, University of North Carolina, Chapel Hill, NC USA

[5] Department of Pulmonary Medicine, The University of Texas MD Anderson Cancer Center, Houston, TX USA

[6] Covance Laboratories Ltd. Woolley Road, Alconbury, Huntingdon, PE28 4HS UK

Results

Pharmacokinetics of ETX004 in mice

In parallel with the intraperitoneal dosing of ETX004 described in the main manuscript and illustrated in Figure 3C, a separate group of mice were dosed with ETX004 (1 mg/kg) by intravenous injection. The concentration of ETX004 in blood over time is shown in Figure S1 from which blood clearance and Vss were calculated to be 40 mL/min/kg and 3.5 L/kg respectively.

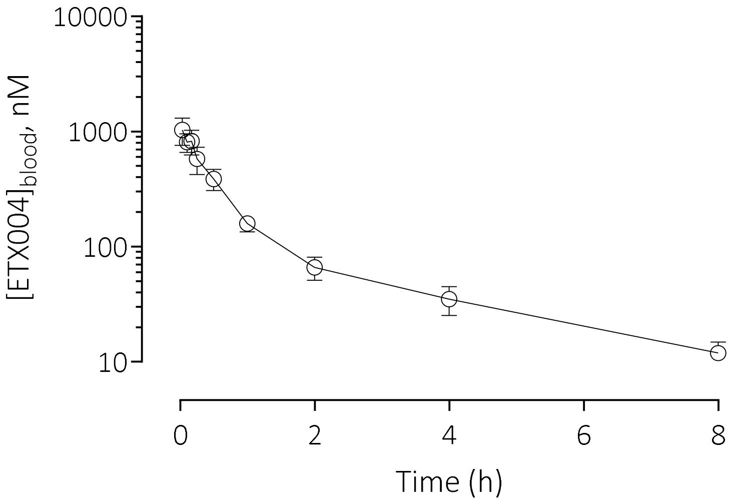

Figure S1 Pharmacokinetics of ETX004 following a single intravenous dose in mice

ETX004 was dosed by intravenous injection (1 mg/kg) to male C57bl6 mice and serial blood samples were collected and analysed for concentrations of test item. Mean data ± SEM are shown (n=3 mice).

Effects of TMEM16A modulators on ciliated cells

Ciliated cell staining using an anti-acetylated α-tubulin antibody was evaluated in the same experiments illustrated in Figure 4, showed no influence of any of the treatments on the ciliated cell population in HBE (Figure S2). Although extended treatment of HBE with IL-13 can reduce the numbers of ciliated cells (1), in the present study where the epithelium was already differentiated prior to IL-13 exposure, there was no effect on the ciliated cell population. Similarly, neither ETX001 or Ani9 affected ciliated cell staining under either naïve or IL-13 stimulated conditions.

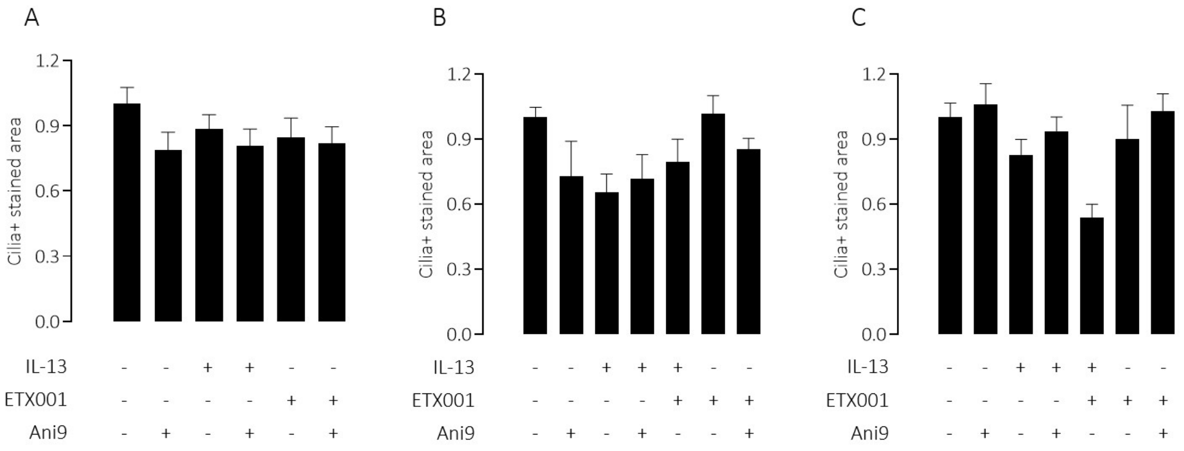

Figure S2 Pharmacokinetics of ETX004 following a single intravenous dose in mice

Acetylated α-tubulin positive ciliated cells were quantified in 3 independent codes of non-CF HBE following treatments with IL-13, Ani9 (10 µM) or ETX001 (1 µM). Panels (A-C) illustrate mean acetylated α-tubulin^{+} stained area ± SEM (n≥4 inserts per group) normalized to the untreated control for each of the 3 donor codes. A one-way ANOVA with post-hoc Sidak test was used to test for significant differences between groups.

Supplemental references

1. Danahay H, Pessotti AD, Coote J, et al. Notch2 is required for inflammatory cytokine-driven goblet cell metaplasia in the lung. *Cell Rep.* 2015;10(2):239-52.
